# *In utero* exposure to HIV and/or antiretroviral therapy: a systematic review of preclinical and clinical evidence of cognitive outcomes

**DOI:** 10.1002/jia2.25275

**Published:** 2019-04-15

**Authors:** Megan S McHenry, Kayode A Balogun, Brenna C McDonald, Rachel C Vreeman, Elizabeth C Whipple, Lena Serghides

**Affiliations:** ^1^ Department of Pediatrics Indiana University School of Medicine Indianapolis Indiana USA; ^2^ Academic Model Providing Access to Healthcare (AMPATH) Eldoret Kenya; ^3^ Toronto General Hospital Research Institute University Health Network Toronto Canada; ^4^ Department of Radiology and Imaging Sciences Indiana University School of Medicine Indianapolis Indiana USA; ^5^ Ruth Lilly Medical Library Indiana University School of Medicine Indianapolis Indiana USA; ^6^ Department of Immunology Institute of Medical Sciences Toronto Canada

**Keywords:** antiretroviral therapy, brain, cognition, highly active, HIV, laboratory animal science, maternal exposure

## Abstract

**Introducion:**

With the increasing number of children exposed to HIV or antiretroviral therapy *in utero,* there are concerns that this population may have worse neurodevelopmental outcomes compared to those who are unexposed. The objective of this study was to systematically review the clinical and preclinical literature on the effects of *in utero* exposure to HIV and/or antiretroviral therapy (ART) on neurodevelopment.

**Methods:**

We systematically searched OVID Medline, PsycINFO and Embase, as well as the Cochrane Collaborative Database, Google Scholar and bibliographies of pertinent articles. Titles, abstracts, and full texts were assessed independently by two reviewers. Data from included studies were extracted. Results are summarized qualitatively.

**Results:**

The search yielded 3027 unique titles. Of the 255 critically reviewed full‐text articles, 25 met inclusion criteria for the systematic review. Five articles studied human subjects and looked at brain structure and function. The remaining 20 articles were preclinical studies that mostly focused on behavioural assessments in animal models. The few clinical studies had mixed results. Some clinical studies found no difference in white matter while others noted higher fractional anisotropy and lower mean diffusivity in the brains of HIV‐exposed uninfected children compared to HIV‐unexposed uninfected children, correlating with abnormal neurobehavioral scores. Preclinical studies focused primarily on neurobehavioral changes resulting from monotherapy with either zidovudine or lamivudine. Various developmental and behavioural changes were noted in preclinical studies with ART exposure, including decreased grooming, decreased attention, memory deficits and fewer behaviours associated with appropriate social interaction.

**Conclusions:**

While the existing literature suggests that there may be some neurobehavioral differences associated with HIV and ART exposure, limited data are available to substantially support these claims. More research is needed comparing neurobiological factors between HIV‐exposed uninfected and HIV‐unexposed uninfected children and using exposures consistent with current clinical care.

**Abbreviations**
ART: anti‐retroviral therapy
BDNF: brain‐derived neurotrophic factor
DTI: diffusion tensor imaging
FA: fractional anisotropy
HEU: HIV‐exposed, but uninfected
HIV: human immunodeficiency virus
HUU: HIV‐unexposed, uninfected
IQ: intelligence quotient
MRI: magnetic resonance imaging
NMDA: N‐methyl‐D‐aspartate

## 1 Introduction

For the 1.4 million children born annually to mothers living with HIV, there are concerns that HIV or antiretroviral therapy (ART) exposure may negatively impact neurodevelopment, including cognition, language and motor skills 1, 2. A meta‐analysis on neurodevelopment in HIV‐exposed uninfected (HEU) children highlighted the limitations in the current body of literature including the heterogeneity of the patient populations between studies, limited confounders measured and variability in ART regimens. More recent studies are often limited by sample size and have mixed results 3, 4, 5.

There are inherent challenges in studying the effects of HIV and ART on neurodevelopment. Children's brains undergo rapid growth and restructuring from birth to adolescence, so functional expressions of neurodevelopmental systems damaged *in utero* may not be easily detectable early in life or may not manifest until later 6. For example, most assessments performed on young children measure general categories of neurodevelopment, such as overall cognitive ability. This prevents more refined analysis of processing speed, working memory and fluid reasoning, which can be measured with greater psychometric rigor when children are older. An additional challenge is delineating the direct effects of HIV exposure compared to ART exposure. Prospectively evaluating HIV exposure *in utero* without ensuring the pregnant mother is also on ART is unethical. However, this delineation could be critical if HEU children were found to have worse neurodevelopmental outcomes.

To overcome some of these challenges, there is a need for both clinical studies using highly sensitive and quantifiable tools and preclinical studies controlling biological states, to delineate the effects of HIV from ART. The objective of this study was to systematically review the clinical and preclinical literature on the effects of *in utero* exposure to HIV and/or ART on neurodevelopment.

## 2 Methods

### 2.1 Search strategy

We conducted a systematic search using a protocol designed by a medical librarian (EW) in accordance with PRISMA guidelines 7. Ovid MEDLINE, PsycINFO and Embase were searched on 17 October 2017 using a comprehensive search strategy (Table S1). On 20 January 2018, we searched Google Scholar, Cochrane Database for Systematic Reviews and the bibliographies of pertinent articles.

The initial screening of titles and abstracts was performed by two independent reviewers (MM and KB). Articles were excluded if they did not include a population exposed to either ART or HIV *in utero* or did not look at a neurological outcome. Full texts of the remaining articles were independently reviewed (MM and KB) to determine whether articles met the complete predetermined eligibility criteria, with disagreements between the reviewers settled after discussion.

### 2.2 Eligibility

Inclusion criteria: 1) a population either exposed to ART *in utero* or exposed to HIV *in utero* without contracting the virus, and 2) primary outcome was an objective measure of neurological or cognitive status. For clinical studies, which inherently involve human subjects, this included: measuring brain structure, brain response, or neurobiomarkers with or without a neurodevelopmental assessment. An emphasis was placed on more quantitative measures of brain structure, response and neurobiomakers, in order to minimize variation in the interpretation of neurodevelopmental assessments alone, which primarily assess behaviour that can be culturally dependent and often use different scales for standardization. For preclinical studies involving animal models, this included: measuring brain structure, brain response, neurobiomarkers or neurodevelopmental assessment. Additionally, for preclinical rodent studies, exposure to ART or HIV‐related proteins prior to postnatal day 7 was considered prenatal exposure, consistent with known benchmarks of neurological maturation between human and rodent foetuses 8. Exclusion criteria: studies that only focused on HIV‐infected populations or used HEU populations only as a control; studies that only measured neurodevelopment in humans (due to the existence of prior related reviews and known heterogeneity in quality of assessments); review articles; published abstracts without full‐text publications; and case study reports containing < 5 participants.

### 2.3 Quality assessment

Quality of clinical and preclinical studies was assessed using The Strength of Evidence Tool 9 and the Animal Research: Reporting of *In Vivo* Experiments (ARRIVE) guidelines 10 respectively. Reviewers independently rated each article, and disagreements were settled after discussion by consensus (Tables S2 and S3).

### 2.4 Data extraction

Study data were extracted into an electronic table by one reviewer (MM) and cross‐checked independently by the second reviewer (KB), including study design, study population/model organism, exposures, neurological/cognitive/biological outcomes measured, main results and limitations. Data were described qualitatively.

## 3 Results

**Figure 1:**
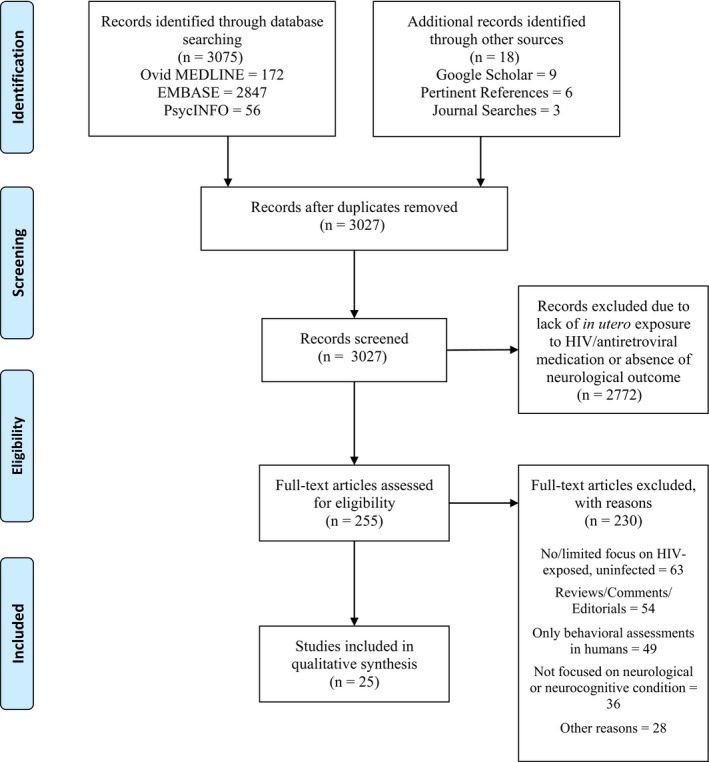
PRISMA flowchart

The searches yielded 3027 unique titles. Post‐screening, 255 full‐text articles were critically reviewed, and 25 met inclusion criteria for the systematic review (Figure 1). Five articles included human subjects and studied brain size, structure and function 11, 12, 13, 14, 15. The remaining 20 articles were preclinical studies using animal models mostly focused on behavioural assessments (see Tables 1 and 2 for study characteristics and summary of outcomes).

**Table 1:** Clinical characteristics

Author (year)	Country	Study design	Study population (clinical)	Exposures	Biological outcome	Clinical neurodevelopmental outcomes	Main outcomes	Limitations
Jahanshad (2015) 11	Thailand	Cross‐sectional	n = 30 HEU n = 33 age‐matched HUU Ages: five to fifteen years	*In utero*: HIV ART exposure only in those born after 2000 (regimen unknown) Infant: Some received AZT prophylaxis	Brain Magnetic Resonance Imaging with Diffusion Tensor Imaging	Wechsler Preschool and Primary Scale of Intelligence‐III Wechsler Intelligence Scale for Children‐III	No volumetric differences or differences in white matter integrity or brain structure between HEU and HUU. The diffusion tensor imaging measures were associated with Full Scale IQ and Performance IQ scores, but not Verbal IQ scores.	Small sample size and no power calculations.
Jankiewicz (2017) [12]	South Africa	Prospective Cohort	n = 65 HIV+ (51 with ART prior to 12 weeks of age, 14 with ART after 12 weeks) n = 19 HEU n = 27 HUU All were seven years old	*In utero*: HIV ART exposure for HIV+ infants varied between two regimens: single dose NVP alone or with AZT after 34 weeks gestation ART exposure for HEU was unknown *Infant:* Early versus late ART for HIV+ only	Brain Magnetic Resonance Imaging with Diffusion Tensor Imaging	n/a	In HEU children compared to HUU a cluster in the right posterior corona radiata with higher fractional anisotropy and increases and decreases in diffusivity metrics was found, while bilateral regions in the corticospinal tract demonstrated reduced diffusivity	Small sample size and no power calculations. Cohort was not assessed for prenatal or perinatal HIV infection.
Poblano (2004) 13	Mexico	Prospective Cohort	n = 37 HEU (12 AZT alone, 25 AZT/3TC) n = 37 HUU Age of HEU: 40.31 ± 6.72 weeks Age of HUU: 42.56 ± 4.79 weeks	*In utero*: HIV 12 AZT alone, 25 AZT/3TC	Brainstem auditory evoked potentials	n/a	Comparison of wave latencies showed significant delay of wave I and I‐III interwave interval in the AZT‐3TC treated group (*p* < 0.05). This subclinical effect on the auditory pathway would affect lower brainstem function.	Small sample size and no power calculations. Short follow‐up period.
Tardieu (2005) 14	France	Retrospective chart review	n = 49 HEU Ages: 10 to 44 months	*In utero*: HIV AZT (some as monotherapy, others with AZT included in various combinations)	Brain magnetic resonance imaging	Neurological assessment	50% of MRIs were abnormal with diffuse hyperintensity in the supratentorial white matter (n = 13) and in the pontine tegmentum (n = 14) as the most common findings. Other children had cerebral volume loss (n = 10), abnormality in the basal ganglia (n = 4), and some had necrosis in the white matter (n = 3).	Patients were selected based on being symptomatic, biasing the sample towards abnormality.
Tran (2016) 15	South Africa	Cross‐sectional study nested within an observational study	n = 15 HEU n = 24 HUU Ages: two to four weeks	*In utero*: HIV Triple therapy ART (not defined)	Brain Magnetic resonance imaging with diffusion tensor imaging	Dubowitz neurobehavioral scale	HEU were found to have higher fractional anisotropy in the middle cerebellar peduncles compared to HUU neonates, after correction for age and sex. Scores on the Dubowitz abnormal neurological signs subscales were positively correlated with fractional anisotropy (r = 0.58, *p* = 0.038) in the left uncinate fasciculus in HEU infants and negatively correlated with diffusivity metrics in the right inferior cerebellar peduncle and bilateral hippocampal cingulum in HEU infants.	Small sample size and no power calculations. Unclear how these results will translate over time.

**Table 2:** Preclinical study characteristics

Author (year)	Country	Study design	Model organism (preclinical)	Exposures	Biological outcome	Phenotypic neurodevelopmental outcomes	Main outcomes	Limitations
Applewhite‐Black (1998) 16	United States	Prospective cohort	Sprague‐Dawley Rat model with two groups n = 30 pregnant dams (16 in AZT group, 14 in vehicle (saline)	*In utero*: Vehicle (saline) only AZT 150 mg/kg/day Treatment and vehicle both given by gastric intubation	n/a	Developmental milestones Growth effects Amphetamine challenge at PND 21 (saline, 0.25, 0.5, or 1 mg/kg dose of amphetamine)	AZT exposed male pups exhibited pinna detachment two days before the vehicle group (*p* = 0.004), no other groups differences noted. AZT exposure significantly reduced litter size and increased birth weights for both male and female pups. AZT exposure increased the locomotor response to amphetamine in females only (*p* < 0.01) and dampened the action of amphetamine to decrease wall hugging in both sexes.	The pups were not fostered by non‐treated dams at birth, so some of the prenatal treatment effects may have contributed to maternal behaviours that altered the pup's behaviour.
Barks (1993) 17	United States	Cohort	Rat model (type not disclosed) n = 67 rat pups	Pups: Excitatory amino acid agonist N‐methyl D asparatate (NMDA) alone (5 nmol) NMDA with coinjection of HIV‐derived recombinant fusion peptide envelope gag (env‐gag) (50 ng env‐gag; 100 ng env‐gag) Intracerebral injections of either NMDA and NMDA/env‐gag at PND 7	Histopathology scoring and measurements of hippocampal cross‐sectional areas on PND 12	n/a	Coinjection of 100 ng of env‐gag with 5 nmol of NMDA markedly increased the severity of resulting injury (*p* < 0.002 for histopathology scores; *p* < 0.003 for interhemispheric differences in hippocampal areas).	Challenges in determining the concentrations of the peptide attained in the brain and the duration of exposure.
Busidan (1999) 18	United States	Cohort	Sprague‐Dawley Rat model with five groups n = 100 to 135 pups per treatment group	*In utero*: Vehicle only AZT 50 mg/kg AZT 100 mg/kg AZT 150 mg/kg No treatment Treatment and vehicle given by gastric intubation for vehicle/treatment mothers and pups. The group that received non‐treated control group was not intubated. Infant: Same exposures as *in utero*	n/a	After injection of amphetamine (0.25, 0.50, 0.75, or 1.0 mg/kg), placed in a Digiscan Activity Monitor box for 60 minutes of behavioural recording	Perinatal AZT exposure alters behaviour in a single domain, locomotion, with males in the AZT 150 group displaying the greatest amount of locomotion while among the females, the AZT 50 group was the most active. Across all treatment groups, amphetamine increased locomotion, the duration of rearing, and sniffing, while it decreased wall hugging, grooming and time spent quiet.	Only the AZT‐induced effects on locomotion were independent of the effects of handling. Thus, daily handling and intubation procedures may have affected several behaviours of the rats.
Calamandrei (1999a) 19	Italy	Cohort	CD‐1 mouse model with four groups n = 12 litters in each group	*In utero*: 0.2 mg/ml AZT 0.4 mg/ml AZT 2.0 mg/ml AZT No treatment Treatment given orally through drinking water. Mice received fresh vehicle or AZT solution four days after sterilization of the drinking bottles.	n/a	Assessment of Somatic and Neurobehavioral Development (PNDs 2 to 20) Locomotor Activity (PND 21) Passive Avoidance Learning and Retention (PNDs 22 and 90) Social‐Aggressive Interaction (PND 41) Gender Preference (PND 83) Intermale Aggressive Behaviour (PND 150)	Male pups receiving 0.4 mg/mL dose of AZT showed a delayed maturation of pole grasping response (*p* = 0.047). Locomotor activity, sex preference, and intermale aggressive behaviour were not significantly influenced by AZT. Intermale aggressive behaviours tended to be increased in frequency in AZT exposed mice compared to controls, but again, this was not statistically significant. AZT appeared to induce a slight impairment during the acquisition session of the passive avoidance task in prepuberty (*p* = 0.022). This result was also seen in young adult mice treated with 0.4 mg/ml AZT (*p* = 0.084). No differences between AZT exposed and controls on retention. AZT treated mice had less digging (*p* < 0.05) and higher number of aggressive bouts (*p* < 0.05) compared to controls, specifically with the 0.2 mg/ml AZT‐treated mice. There was a tendency towards more intermale aggression, but it was not statistically significant.	Offspring's viability was severely affected in the 2.0 mg/ml group, so all analyses were only with the remaining groups. Because the treated mice were received the AZT solution via drinking water, the exact AZT dosing was not standardized among each group of mice.
Calamandrei (1999b) 20	Italy	Cohort	CD‐1 mouse model with three groups n = 10 litters in each group	*In utero*: 0.4 mg/ml AZT (corresponding to 80 mg/kg/day) 0.8 mg/ml AZT (corresponding to 160 mg/kg/day) No treatment Treatment given orally through drinking water. Mice received fresh vehicle or AZT solution four days after sterilization of the drinking bottles.	n/a	Passive avoidance learning and retention Acquisition‐ PND 15 Retention‐ PND 16 Morris Water Maze Test (PNDs 45 to 50) for spatial learning	Pups learned the avoidance response regardless of the exposure group, but the number of trials needed to reach the learning criterion tended to be higher in the group exposed to 0.8 mg/ml AZT (*p* = NS). Retention was lower in the 0.8 mg/ml AZT group compared to the control (*p* = 0.041) and 0.4 mg/ml AZT (*p* = 0.014) groups No treatment effects were found for spatial learning.	
Calamandrei (1999c) 21	Italy	Cohort	CD‐1 mouse model with four groups n = 12 litters in each group	*In utero*: 3TC (125 mg/kg) 3TC (250 mg/kg) 3TC (500 mg/kg) Vehicle/Saline Vehicle or 3TC given per os twice daily	n/a	Somatic and Neurobehavioral Development (PNDs 2 to 18) Ultrasonic Vocalizations (PNDs 3, 7 and 11) Passive Avoidance Learning and Retention (PND 20) Locomotor Activity (PND 22)	No gross changes in somatic and sensorimotor development. 3TC exposure did not affect learning and retention performances of a passive‐avoidance task. A slight decrease in ultrasound emission was found on PND 3, in 125 and 500 mg/kg 3TC exposed groups compared to the 150 mg/kg 3TC and control groups (*p* < 0.05). This was not present on PNDs 7 and 11. The pups learned the avoidance response regardless of prenatal treatment received. However, the effect of 3TC on the number of trials needed to reach the criterion approached statistical significance (*p* = 0.066). Decreased habituation in an automated locomotor activity test was present in males within the 250 and 500 mg/kg 3TC groups.	
Calamandrei (2000a) 22	Italy	Cohort	CD‐1 mouse model with two groups n = 9 litters in each group	*In utero*: AZT/3TC (160 and 500 mg/kg dose) Vehicle/Control Vehicle or treatment given orally, twice daily	n/a	Open field and scopolamine challenge with behaviour categories analysed by “The Observer” software	AZT/3TC exposure did not influence responsiveness to the muscarinic cholinergic antagonist as measured by analysis of the drug's effects on locomotor and exploratory activity and different behavioural items. AZT/3TC‐treated mice displayed higher frequency of rearing (*p* < 0.05), and lower frequency and duration of self‐grooming behaviour (*p* < 0.05).	Only used a single dose of scopolamine.
Calamandrei (2000b) 23	Italy	Cohort	CD‐1 mouse model with four groups n = 12 litters in each group	*In utero*: 3TC (125 mg/kg) 3TC (250 mg/kg) 3TC (500 mg/kg) Vehicle/Control Vehicle or 3TC given orally, twice daily	n/a	Social interaction –PND 35 (non‐social and social responses) Open field and scopolamine challenge at PND 60 Spatial Learning at PND 90 – Morris water maze and radial eight‐arm maze Pain sensitivity at PND 90 – Hot‐plate test Maternal behaviour induction at PND90	3TC exposure was associated with a decrease in immobility in the open field test, an increase in the responsiveness to scopolamine in an open field (500 mg 3TC only), and a longer escape latency in the first day of the reversal phase in the Morris task (particularly in the 250 mg/kg 3TC group). Social interactions, radial arm maze, and the hot‐plate test did not exhibit any treatment effects. Higher risk of cannibalism was found in 3TC exposed female groups (especially in the 125 mg/kg and 500 mg/kg) groups compared to the control and 250 mg/kg 3TC groups (*p *= NS).	Experiments performed on small numbers of the overall sample.
Calamandrei (2002a) 24	Italy	Cohort	CD‐1 mouse model with two groups n = 24 litters in each group	Experiment 1: *In utero*: 160 mg/kg/day AZT Vehicle/Control Vehicle or AZT given orally, twice daily, from gestational day 10 to 19 Experiment 2: *In utero*: 160 mg/kg/day AZT Vehicle/Control Vehicle or AZT given orally, twice daily, from gestational day 10 to lactation day 10 Pup: after delivery, the pups nursed from the mother in their respective treatment group until lactation day 10	n/a	Behavioural procedure using open field‐ locomotor activity and other behavioural responses noted on PND 28, 45, and 70.	Experiment 1: AZT exposure reduced exploration of the objects at all ages considered (*p* < 0.01) and increased wall and top rearing at PND 45 (*p* < 0.05) Experiment 2: AZT‐exposed offspring were more active than controls and AZT‐exposed males displayed more wall rearing at age PND 70 (*p* < 0.05). AZT exposure was associated with lower grooming frequency at all ages (*p* < 0.05).	
Calamandrei (2002b) 25	Italy	Cohort	CD‐1 mouse model with two groups	*In utero*: 160 mg/kg/day AZT Vehicle/Control Vehicle or AZT given orally, twice daily from gestational day 10 to lactation day 7 Pup: after delivery, the pups nursed from the mother in their respective treatment group until lactation day 7	Brain‐derived neurotrophic factor (BDNF) Nerve growth factor (NGF) Both BDNF and NGF were measured at PND 7, 21, and 60	n/a	BDNF levels were increased in the parietal cortex for both males and females exposed to AZT throughout the time points. In AZT‐exposed females, BDNF levels were also increased in the hippocampus on days 7 and 21 (*p* = 0.0062) and in the hypothalamus on day 21 (*p* = 0.008). There were no changes in NGF in AZT‐exposed females in the cortex, hypothalamus or striatum. In AZT‐exposed males, there were no other statistically significant difference in BDNF and NGF compared to unexposed males.	Unclear sample size
Fitting (2008) 26	United States	Cohort	Sprague‐Dawley rats n = male pups from 13 litters	*In utero*: None Pup: on PND 1, pups were given bilateral intrahippocampal injections of the following treatments:	At 7.5 months of age, the total number of cells were quantified in the five hippocampal subregions: granule layer, hilus of the dentate gyrus, cornu ammonis fields, CA1 in the cornu ammmonis, and subiculum This was done to test the relationship between behaviour and anatomy	Early reflex development: righting reflex (PND 3 to 5), negative geotaxis (PND 8 to 10) Sensorimotor function (PND 18 and 91) Locomotor activity (PND 21 to 23 and 94 to 96) Spatial learning and memory (PND 49 to 55 and 113 to 121) (Acquisition training and probe test)	Tat protein had an overall transient effect on many of the behavioural assessments early in development. Tat also had an effect on preattentive processes and spatial memory in adulthood. gp120 had more selective effects on negative geotaxis (PND 8 to 10) and locomotor activity (PND 94 to 96). A relationship between early reflex development and estimated cell numbers in the hippocampus was indicated. Estimated number of neurons and astrocytes in the hilus of the dentate gyrus explained 81% of the variance of the distribution of searching behaviour in the probe test of spatial memory.	
Levin (2003) 27	United States	Cohort	CD‐1 mouse model with three groups n = 9 to 10 per sex per treatment group	*In utero*: AZT 100 mg/kg/day AZT 200 mg/kg/day Control/Vehicle Treatment given by gavage twice daily	n/a	Elevated plus maze‐ to test anxiety Radial‐arm maze‐ to test spatial learning and memory (with intra‐session delays of 90 seconds, 15 minutes, 2.5 hours, and 4 weeks) Balance beam‐ to test vestibular‐moto performance	There was no significant effect of AZT treatment on radial‐arm maze, and introducing an intra‐session delay of 90 seconds, 15 minutes, or 2.5 hours did not alter performance. After the four week intra‐session, locomotor activity on the radial‐arm maze was significantly affected by AZT treatment (100 mg/kg/day) during the acquisition phase (*p* < 0.05), but not during the other test phases. This effect was in the direction of improved performance relative to controls. No significant effects of AZT treatment on plus maze or balance beam.	
Melnick (2002) 28	United States	Cohort	Sprague‐Dawley rats n = No treatment (14 litters) Vehicle (12 litters) AZT 50 mg/kg/day (11 litters) AZT 100 mg/kg/day (12 litters) AZT 150 mg/kg/day (9 litters)	*In utero*: No treatment Vehicle AZT 50 mg/kg/day AZT 100 mg/kg/day AZT 150 mg/kg/day Treatment given once daily by gastric intubation	n/a	Acoustic startle response – testing between PND 75 to 80 following a challenge of either saline or 1.0 mg/kg amphetamine intraperitoneally	The AZT 100 mg/kg group had increased acoustic startle response habituation. AZT treatment did not affect pre‐pulse inhibition. Females in the AZT 150 mg/kg group had high acoustic startle responses at the end of the startle session (*p* < 0.008). AZT‐treated animals showed a dose‐dependent increase in peak latency (*p* < 0.05), suggesting a possible abnormal conduction velocity.	
Melnick (2005) 29	United States	Cohort	Sprague‐Dawley rats n = No treatment (8 litters) Vehicle (8 litters) AZT 100 mg/kg/day (8 litters) AZT 150 mg/kg/day (8 litters)	*In utero*: No treatment Vehicle AZT 100 mg/kg/day AZT 150 mg/kg/day Pups: Received same treatment as mother from PND 2 to 20 Treatment given once daily by gastric intubation for gestational day 19 to 22 for pregnant mice	n/a	Acoustic startle response and tactile stimuli response, performed at PND 60 following a challenge of either a vehicle, 0.25 or 0.5 mg/kg 8‐OH‐DPAT (serotonin agonist) OR 0.75 or 2.0 mg/kg apomorphine (APO, a dopaminergic agonist), intraperitoneally	Perinatal AZT exposure enhanced startle responses following both DPAT and APO. Perinatal AZT increased tactile responses following drug challenge, although magnitude of the increase was dependent on AZT exposure level and gender. Perinatal AZT also prolonged startle latencies (*p* = 0.013), a change which may indicate that perinatal AZT alters conduction velocity.	
Morton (1993) 30	United States	Cohort	Pigtailed macaques n = 7 offspring from SIV‐infected mothers (5 of which were exposed to SIV but uninfected)	*In utero*: SIV intravenously given either in the second or third trimester	SIV antigens	Object permanence testing Wisconsin General Testing Apparatus (WGTA) – cognitive testing (black/white discrimination and reversal, Hamilton search	Object permanence – 2/3 of the animals SIV‐exposed in the second trimester reached criterion later than colony normal. Both animals exposed to SIV in the third trimester did poorer than colony norms on a specific cognitive test (forced set breaking), one of the two significantly poorer than colony norms on multiple specific cognitive tasks.	Very small sample size
Ricceri (2001) 31	Italy	Cohort	CD‐1 mouse model with three groups n = 8 litters from each of the three original treatment groups of 24 litters (for each of the three exposure groups) were assigned to testing at three different postnatal ages (PND 8, 14, and 28)	*In utero*: Vehicle/Saline AZT 160 mg/kg/day 3TC 500 mg/kg/day Treatment or vehicle was given orally twice daily from pregnancy day 10 to delivery	n/a	Locomotor Activity (using a Varimex Activity apparatus) at PND 8, 14, and 28 after administration of GABA receptor agonist muscimol Hot‐plate Test	At PND 8, high dose muscimol was associated in increased locomotor activity in AZT‐ and 3TC‐exposed mice. At PND 14, low muscimol dose enhanced locomotor activity in vehicle and 3TC but not in AZT‐exposed pups. At PND 28, no prenatal treatment effect was seen in locomotor activity. AZT increased nociceptive sensitivity at all time points, especially in female pups.	
Rondinini (1999) 32	Italy	Cohort	CD‐1 mouse model with three groups n = 20 males from 10 litters per treatment group (two subjects per litter), 60 males total	*In utero*: Vehicle/Saline AZT 0.4 mg/ml (corresponding to 80 mg/kg/day) AZT 0.8 mg/mL (corresponding to 160 mg/kg/day) Given in drinking water to female mice from gestational day 10 to delivery	n/a	Inter‐male aggressive behaviour at PND 90 and PND 150	At PND 90, only slight changes in both aggressive and defensive components of male specific agonistic pattern, with AZT‐exposed mice having a limited increase of aggressive behaviour compared to controls, specifically during the following behavioural items: *Chase, Offensive Upright Posture, Tail Rattling,* and *Defensive Upright Posture*. There was a decrease is specific time intervals of *Escape* and *Upright Submissive Posture*. At PND 150, no exposure effects were found.	Because the treated mice were received the AZT solution via drinking water, the exact AZT dosing was not standardized among each group of mice.
Venerosi (2001) 33	Italy	Cohort	CD‐1 mouse model with two groups n = 12 litters in each group	*In utero*: AZT/3TC (160/500 mg/kg dose) Vehicle/Control Treatment or vehicle was given orally, twice daily, from gestational day 10 until delivery	n/a	Somatic and neurobehavioral development (PND 2 to 18) Homing test (PND 10) Passive‐avoidance acquisition and retention (PND 22 to 23) Locomotor activity (PND 23) Social interaction (PND 35)	The AZT/3TC‐exposed group had slightly delayed maturation of forelimb placing (*p* = 0.0095), forelimb stick grasping (*p* = 0.05), level screen (*p* = 0.0093), and pole grasping (*p* = 0.0038). No effects on passive‐avoidance, homing test, or locomotor activity were found. AZT/3TC mice showed selective alterations in the social interaction test, and the females also displayed a significant reduction of affiliative interactions, such as mutual circling (*p* = 0.0325) and allogrooming (*p* = 0.0055).	
Venerosi (2005) 34	Italy	Cohort	CD‐1 mouse model with two groups n = 60 male offspring	*In utero*: 160 mg/kg/day AZT (for gestational day 10 to 19) Vehicle/Control	n/a	Automated activity test for grooming, wall rearing, and locomotion At PND 60, received intraperitoneal injection of D1 receptor agonist SKF 38,393 20 minutes prior to automated activity test. Doses used were 0, 3, and 10 mg/kg.	No significant difference in grooming between AZT and control groups. However, as the challenge dose of SKF 38,393 increased, only the control mice had the expected increase in grooming duration. There was no significance difference between control and AZT groups for wall rearing, rearing, or locomotion for either dose of the D1 agonist.	
Zuena (2013) 35	Italy	Cohort	CD‐1 mouse model with four groups n = 45 litters were randomly assigned to a group	*In utero*: Control/vehicle L‐acetylcarnitine (LAC) (LAC subQ and water, orally) AZT (saline subQ+AZT orally) ZT+LAC (LAC subQ+AZT orally)	Expression of iGlu and mGlu in the hippocampus, determined by western blot analysis Corticosterone secretion after acute restraint stress (results not reported here)	Water Maze Procedure (spatial learning and memory)	AZT administered during gestation did not reach detectable levels in the plasma of pups, but significant AZT levels were found in the brain, indicating transplacental passage (*p* < 0.05) The AZT+LAC group had escape latencies of spatial learning and memory comparable to the control group and significantly different from those of the AZT group in the third and fourth sessions (*p* < 0.05). The AZT group had a significant reduction of mGlu1a and mGlu5 receptor expression compared to the control (*p* < 0.05). The mGlu1a and mGlu5 receptors in the AZT‐LAC group showed a trend to increase compared to AZT, but was not statistically significant.	

LAC, L‐acetylcarnitine; OH‐DPAT, hydroxyl‐2‐(diprophylamino)‐tetralin; PND, Postnatal day; SIV, Simian Immunodeficiency Virus.

### 3.1 Clinical studies

Four studies looked at magnetic resonance imaging (MRI) findings in HEU populations; two were performed in South Africa 12, 15, one in France 14 and one in Thailand 11. In terms of methodological quality, one study was rated as good 12, two were fair‐good 13, 15 and two were fair 11, 14. Three studies used diffusion tensor imaging (DTI) 11, 12, 15. Two noted regionally higher fractional anisotropy (FA) and predominantly lower diffusivity in HEU compared to HUU, in both neonates and children 12, 15. Higher FA was noted in the middle cerebellar peduncles and right posterior corona radiata 12, 15, and lower diffusivity was noted in bilateral regions of the corticospinal tract, while the posterior corona radiata showed both significant increases and decreases in different diffusivity metrics 12. Abnormal Dubowitz neurobehavioral scores were positively correlated with FA in the left uncinate fasciculus and negatively correlated with diffusivity in the right inferior cerebellar peduncle and bilateral hippocampal cingulum in HEU infants 15. The third study using DTI in age‐matched HEU and HUU children did not detect group differences in intelligence quotient (IQ) scores, brain volume, or DTI metrics, after controlling for sociodemographic factors 11. This study found that DTI measures were significantly associated with full scale and performance IQ scores, showing positive associations with FA and negative associations with diffusivity metrics. Subscale analyses showed the strongest effects between perceptual organization scores and diffusivity in the internal capsule, cingulum, and optic/temporal regions, including the uncinate and thalamic radiations 11.

The fourth MRI study only looked at HEU children (n = 49) and found that 50% showed MRI abnormalities, including diffuse hyperintensity in the white matter and pontine tegmentum 14. Additionally, cerebral volume loss was seen in 10 children 14. This study was a retrospective chart review of HEU infants and toddlers, the majority (88%) of whom were symptomatic, introducing sample bias towards abnormality 14.

One study looked at brainstem auditory evoked potentials, and showed significant delays of wave I and I‐III interwave intervals in HEU infants exposed *in utero* to zidovudine alone or in combination with lamivudine 13. The authors suggested that these findings may indicate toxicity in the lower regions of the brainstem in HEU infants 13.

### 3.2 Preclinical studies

Of the 20 preclinical articles, 15 evaluated exposure to ART monotherapy (either zidovudine or lamivudine) 16, 18, 19, 20, 21, 23, 24, 25, 27, 28, 29, 31, 32, 34, 35, two exposure to combination therapy (zidovudine and lamivudine) 22, 33, two exposure to HIV‐derived proteins 17, 26 and one exposure to Simian Immunodeficiency Virus 30. Thirteen used a CD‐1 mouse model 19, 20, 21, 22, 23, 24, 25, 27, 31, 32, 33, 34, 35, five a Sprague‐Dawley rat model 16, 18, 26, 28, 29, one a pigtailed macaque model 30 and one an undisclosed rat model 17. Sixteen studies looked at behaviour and development, while the remaining evaluated neurological biomarkers or structural differences in the brain. Control groups included vehicle (often saline) (n = 12), both vehicle and no treatment (n = 3), or no treatment (n = 2). All studies were from the United States or Italy.

### 3.3 Zidovudine

#### 3.3.1 Cognition/memory

Two studies reported that zidovudine‐exposed mice (prepubertal and young adult) showed either significant impairment or trends towards impairment during the acquisition session of the passive avoidance task, a memory test 19, 20. However, retention of passive avoidance, spatial learning, and memory were not impacted by zidovudine exposure 20, 27, and when a longer intra‐session delay was introduced in a spatial learning and memory task, zidovudine‐exposed mice (100 mg/kg/day) had improved performance over controls (*p* < 0.05) 27.

#### 3.3.2 Motor skills/nociception

*In utero* exposure to zidovudine alone 19, 31, or with the addition of a dopamine receptor D1 agonist 34, did not affect locomotor activity of offspring. However, zidovudine‐exposed neonatal mice showed increased locomotor activity in response to GABAergic agonist treatment early in life, but not persisting into adulthood 31. Zidovudine‐exposed mice also demonstrated increased nociceptive sensitivity at all ages that was not dependent on GABA‐regulated nociceptive mechanisms 31.

#### 3.3.3 Anxiety/sociability

Studies measuring sociability and anxiety‐related behaviours report mixed findings. When given amphetamine to mimic a stress response, two studies found that zidovudine‐exposed rats did not exhibit developmental delays that prohibited them from reacting to the stress 16, 18. Zidovudine‐exposed rats had less wall‐hugging behaviours (possibly indicative of lower anxiety) and an increased locomotor response, but no difference in rearing or sniffing. The increased locomotor response to amphetamine was only seen in females in one study 16, but in both sexes in the other 18.

One study noted that prenatal exposure to zidovudine was associated with reduced exploration of objects at all ages considered, increased wall‐ and top‐rearing at specific ages, and hyperactivity in adulthood 24. However, another study reported no significant differences in locomotion or rearing between zidovudine‐exposed mice and controls, even after dopamine agonist injection 34.

Two studies had an additional focus on aggressive behaviours. In one, zidovudine exposure was associated with less digging (*p* < 0.05), higher number of aggressive bouts in both sexes (*p* < 0.05), and a tendency towards more inter‐male aggressive behaviours 19. A second study looking at inter‐male aggressive behaviours reported alterations of both aggressive and defensive actions, with zidovudine‐exposed mice having significantly more frequent aggressive behaviours compared to controls in adolescent but not adult mice 32.

Several studies noted lower grooming frequencies in zidovudine‐exposed mice 18, 24, 34. Lower grooming frequency was also seen in zidovudine‐exposed mice following administration of dopamine receptor D1 agonist or amphetamine, which was not the anticipated effect 18, 34.

Exposure to zidovudine *in utero* was associated with a dose‐dependent increase in peak latency in the acoustic startle response and enhanced tactile stimuli response 28, 29. Perinatal zidovudine exposure enhanced startle responses following injection of amphetamine 28, apomorphine and serotonin agonist 29. These results may suggest abnormal nerve conduction velocity and long‐term functional alterations within the startle reflex pathways 28, 29.

#### 3.3.4 Biomarkers

One study measured brain‐derived neurotrophic factor (BDNF) within various areas of the brain at multiple time points 25. In zidovudine‐exposed mice, BDNF levels were increased in the parietal cortex, hippocampus and hypothalamus compared to controls 25. Sex differences in BDNF concentrations were observed in various brain regions, with zidovudine‐exposed females having increased BDNF levels in the hippocampus, cortex and hypothalamus at various time intervals and males having increased BDNF in the cortex at one time interval 25.

#### 3.3.5 Learning/memory interventions

One study hypothesized that L‐acetylcarnitine, an antioxidant and neuroprotective factor, might improve ART‐induced mitochondrial dysfunction and associated neuropathies 35. The study reported zidovudine‐induced impairment of spatial learning and memory that was counteracted by L‐acetylcarnitine treatment (*p* < 0.05). In addition, zidovudine‐exposed mice had reduced expression of hippocampal metabotropic and ionotropic glutamate receptors, which was counteracted by L‐acetylcarnitine administration during pregnancy 35. Of note, while pups exposed to zidovudine *in utero* had undetectable levels of the drug in plasma at birth, they had significant levels of zidovudine in their brain (70.2 ± 6.1 ng/mg) 35.

### 3.4 Lamivudine

#### 3.4.1 Cognition/memory

Studies investigating the impact of lamivudine exposure on cognition and memory reported mixed results. In one study, lamivudine exposure in mice did not appear to affect acquisition and retention performance in passive avoidance tasks, although there was a trend towards lamivudine‐exposed mice requiring more trials to reach acquisition (*p* = 0.066) 21. A study assessing long‐term neurobehavioral effects reported that lamivudine‐exposed mice had a longer escape latency in the reversal phase in the Morris water maze, suggesting impaired reversal learning, but otherwise working and reference memory were not negatively impacted 23.

#### 3.4.2 Motor skills/nociception

One study reported that lamivudine‐exposed mice (regardless of dose) had lower locomotor activity compared to controls (*p* < 0.05) 21. Male mice exposed to the highest lamivudine dose tested showed decreased habituation in the locomotor activity test (*p* < 0.05) 21. Additionally, no gross changes were seen in somatic and sensorimotor development 21.

A study examining the impact of nucleoside analogues on the GABAergic system reported increased locomotor activity in lamivudine‐exposed mice following administration of a GABA receptor agonist at eight days of age, but this effect was not seen later in life 31.

#### 3.4.3 Anxiety/sociability

One study looked at sociability in lamivudine‐exposed mice. Mice exposed *in utero* to lamivudine (500 mg/kg, the highest dose tested) had non‐specific alterations in sensitivity to a muscarinic cholinergic antagonist (scopolamine), showing increased sniffing behaviour (*p* = 0.0161) and decreased immobility (*p* < 0.05) 23. Lamivudine exposure *in utero* also influenced maternal behaviour, with lamivudine‐exposed mothers showing higher rates of aggressive behaviour towards foster pups, manifesting in cannibalism 23.

### 3.5 Combination therapy (zidovudine/lamivudine)

The first of two studies looking at a combination of zidovudine and lamivudine examined the effect of *in utero* exposure on cholinergic muscarinic neuroregulation in adulthood 22. Responsiveness to a muscarinic cholinergic antagonist did not differ significantly between ART‐exposed mice and controls, with similar habituation and response inhibition. However, ART‐exposed mice displayed a higher frequency of rearing activities (*p* < 0.05) and a lower frequency and duration of self‐grooming behaviour (*p* < 0.05) 22, both behaviours that involve the dopaminergic system 36, 37.

The second study examined the effect of *in utero* exposure to a combination of zidovudine and lamivudine on a variety of neurobehavioral endpoints. ART exposure had a small but marked delayed effect on somatic and sensorimotor development, such as forelimb placing (*p* = 0.0025), forelimb stick grasping (*p* = 0.05), level screen (*p* = 0.0093) and pole grasping (*p* = 0.0038) 33. Additionally, ART‐exposed mice had selective alterations in social interaction tests, with females displaying a significant reduction in affiliative interactions, such as mutual circling (*p* = 0.0325) and allogrooming (*p* = 0.0055) 33. There was no effect on passive avoidance or locomotor activity 33.

### 3.6 HIV‐derived proteins or SIV

One study looked at the impact of HIV‐derived peptides on the neurotoxicity of excitatory amino acid agonists. Rat pups were administered the excitatory amino acid agonist N‐methyl‐D‐aspartate (NMDA) intracerebrally with or without recombinant fusion peptide envelope gag (env‐gag) on postnatal day 7, the equivalent of third trimester exposure for a human foetus 17. Histopathological scoring and measurements of hippocampal cross‐sectional areas showed that the env‐gag/NMDA injection rats had significantly more severe brain injury (*p* < 0.003) compared to those with NMDA injections alone 17. A similarly designed study looked at two additional HIV‐1 proteins, Tat and gp120 26. This study found that Tat had an overall transient effect on many behavioural assessments early in development and on pre‐attentive processes and spatial memory in adulthood, while gp120 had more selective effects on negative geotaxis in neonates and locomotor activity in adults 26. This study also assessed the relationship between behaviour and hippocampal anatomy, and found 81% of the variance in spatial memory (searching behaviour in the probe test) was explained by the estimated number of neurons and astrocytes in the hilus of the dentate gyrus 26.

One very small study using pigtailed macaques looked at behaviour in offspring born to SIV‐infected mothers 30. Five of the offspring were exposed but uninfected, and of these, most completed various object permanence and cognitive testing tasks slower than colony norms 30.

## 4 Discussion

This is the first systematic review of clinical and preclinical studies focused on the cognitive implications of *in utero* HIV or ART exposure. The few clinical studies in HEU children have been mixed. Some found no difference in white matter integrity relative to HUU children, while others reported higher FA and lower diffusivity values, correlating with lower neurobehavioral scores. The preclinical data provided a more comprehensive view of differences in brain structure, behaviour, and biomarkers in models exposed to HIV proteins or two antiretrovirals, with some adding information about how the effects of exposure may persist into adulthood.

While the clinical data were limited, they did reveal some intriguing findings for HEU children. In two of the three clinical studies using DTI, regional increases in FA were noted in HEU children, generally reflecting more densely packed axons, greater axon diameter, or greater myelination. While increased fractional anisotropy may represent higher white matter connectivity 38, 39, it has also been observed in pathological conditions, such as autism 40 and attention‐deficit disorder 41. Lower diffusivity values 12 correlating with lower neurobehavioral scores 12, 15 were also seen in HEU children. Lower diffusivity is thought to be related to denser white and gray matter 39. These findings may be due to other potential confounders, such as poor maternal nutritional status and other *in utero* stress, as these studies only adjusted for the children's sex and age. In another study 11, correlations between DTI metrics and cognitive function fell in the more typically expected direction, with better cognitive functioning correlating with higher FA and lower diffusivity. One of the preclinical studies showed that neuronal and astrocyte density in certain areas of the brain accounted for a majority of the distribution in certain behavioural tasks 26. Difference in methodologies used to measure white matter and study participant characteristics (e.g. age) make it difficult to speculate on mechanisms underlying these potential differences between HEU and HUU children and whether HIV and/or ART play a role. More studies measuring white matter changes and their relationship with clinical neurodevelopmental outcomes are needed to determine if differences exist between HEU and HUU. Future research in this area would provide the most benefit by having the following characteristics: an adequately powered sample size in a longitudinal cohort or a cross‐sectional cohort with a large age band; appropriate technical methodologies; and associated clinical neurocognitive assessments. If a relationship between white matter connectivity and axon density, cognitive delays, and HIV and/or ART exposure was confirmed, it might allow us to identify children at risk for neurodevelopmental impairments at an earlier age, enabling timely intensive interventions providing maximal benefits.

Given increasing access, cognitive studies of HEU children should consider including advanced neuroimaging. This will help reconcile apparent discrepancies in the available studies and clarify brain structure and function relationships within this population. If relationships demonstrated in the preliminary work are replicated, these could be very informative in terms of understanding potential later consequences for HEU children. For example, the corona radiata is a white matter structure connecting the brainstem and cerebral cortex, critical for sensorimotor function, while the uncinate fasciculus and hippocampal cingulum are important for learning and memory. Abnormalities in such white matter pathways may therefore have important clinical implications for effective development of such functions.

The preclinical data also revealed some important findings regarding exposure to ART. For both zidovudine‐ and lamuvidine‐exposed rodents, some studies suggested that memory and learning may be impaired at early ages 19, 23, 27. More recent clinical studies with newer ART regimens have not found such deficits in young children 2, 3, 42. Fewer data are available about specific cognitive functions in older HEU children and adolescents, but studies have found a higher frequency of reading and math impairments in HEU children compared to the general population 43. While this review does not clearly identify HIV or ART exposure as being associated with deficits in cognition or memory, it does raise concerns regarding cognitive development in HEU. The inconsistency of results between preclinical data and clinical data in young children using neurodevelopmental assessments should encourage researchers to carefully consider methodologies used to assess neurodevelopment in young children, specifically focusing on high‐quality, culturally and age‐appropriate assessments and the use of technologies such as MRI to best measure cognitive function. Since some preclinical data reveal defects in younger mice that do not persist into adulthood, longitudinal follow‐up of HEU children in future research is merited.

Social behaviours were another area of concern within the pre‐clinical data. Data suggest that ART‐exposed mice displayed less social behaviours, with significantly less affiliative interactions 33, higher number of aggressive bouts 19, 32, or increased rates of maternal cannibalism of pups 23. ART exposure differed between studies, so the aetiology of the findings remains unclear. However, little is known about social behaviours in HIV‐ and ART‐exposed children and adults. Higher rates of autism spectrum disorder have been found in HEU populations; it is therefore hypothesized that mitochondrial dysfunction may be a contributing factor 44. With the growing population of HEU children worldwide, further research on their long‐term cognitive and social functioning is critically important.

Preclinical research has valuable potential to direct research in humans. Animal models allow for mechanistic studies, which yield clearer results compared to efforts in deciphering specific neurobiological pathways in humans. By including preclinical studies, this review further explores potential effects resulting from ART versus HIV exposure. Most preclinical studies investigated the effects of ART monotherapy, specifically zidovudine and lamivudine, on cognitive function, illuminating potential impact from the individual components of many combination therapies. Other preclinical studies used HIV proteins to mimic HIV‐exposure in the absence of ART exposure, deepening the understanding of the interplay between HIV and ART exposure and brain development and function, which is not possible in human studies. For example, one study determined that zidovudine‐exposed pups had high concentrations of the drug in brain, but undetectable levels in plasma 35. Another study found elevated BDNF levels in the parietal cortex of mice exposed to zidovudine *in utero*, with females having particularly high levels 25. These preclinical studies generate important hypotheses to inform the design of clinical studies, such as appropriate anatomical locations for sampling or the possible influence of oestrogen on BDNF. However, because of the limited number of studies within this review, we were unable to disentangle the effects of ART exposure from HIV exposure. Future preclinical research in this area would benefit from choosing assessments that have comparable measures in clinical research, which may be later evaluated. This will strengthen our knowledge of potential infant outcomes of ART or HIV exposure.

This review has some limitations. We did not expand our search criteria to capture clinical studies looking at mitochondrial disorders and microcephaly in HEU children due to the heterogeneity of this literature. This may hinder us from comprehensively describing other conditions impacting neurodevelopment in HEU children. Also, our interpretation of the clinical data within this review is limited by the heterogeneity of the techniques used to determine structural or neurobiological outcomes, as well as the age of the study participants. While we were unable to hypothesize on a specific mechanism for the structural differences present between HEU and HUU children, we believe that the fact that structural differences were found within the studies warrants further investigation into brain structure and its relationship with clinical neurodevelopmental outcomes as children mature. Additionally, this review was limited by the lack of power calculations, small sample sizes, and the absence of point estimate and 95% confidence interval reporting. These statistical issues limited confidence in the reported results and understanding of how the small sample size may have impacted the results. The potential effects of modern ART regimens were not considered, as most preclinical studies were > 15 years old.

## 5 Conclusions

Due to the complexities of cognitive assessments and confounding variables impacting neurodevelopment, the current literature on neurodevelopment in HEU and HUU children does not clearly indicate whether there are differences between these children. This review summarizes objective data from both clinical and preclinical studies. The combined literature suggests possible differences in white matter connectivity in clinical studies and memory and sociability differences in preclinical studies, although the applicability of these data is limited. However, due to the abnormalities in brain structure, function and biochemistry found in HEU compared to HUU, this is an area where more systematic and translational work is needed. Future preclinical studies should consider looking at specific mechanisms of neurobiological changes and use ART exposures and neurobehavioral assessments that harmonize with current clinical standards. More clinical research is needed comparing the neurobiological factors between HEU and HUU, with a focus on domains found to be impacted in preclinical research, such as memory and sociability. In doing this, we will take steps in determining the clinical implications of *in utero* HIV and ART exposure.

## Competing interest

None of the authors have any competing interest to declare.

## Authors’ contributions

All six authors meet criteria for authorship and have made substantial contributions to various facets of the study including study design, data collection and analysis, and writing and editing of the manuscript. MM first conceptualized this systematic review. EW designed the search criteria for each database, with input from MM. MM and KB reviewed all articles and performed data collection. BM, RV and LS provided expert consultation on paediatric neuroimaging, paediatric HIV, and preclinical studies involving ART/HIV exposure respectively. MM wrote the first draft with considerable input from the coauthors. All six authors take responsibility for the reported research, have critically reviewed this final manuscript, approved its submission and take full responsibility for the manuscript.

## Supporting information


**Table S1.** Search strategy: Ovid MEDLINE

**Table S2.** Quality assessment of clinical studies

**Table S3.** Compliance of preclinical study reporting, by Animal Research: Reporting of *In Vivo* Experiments (ARRIVE) criteria
