# Supplementary material for: In utero exposure to HIV and/or antiretroviral therapy: a systematic review of preclinical and clinical evidence of cognitive outcomes
Source: J Int AIDS Soc. 2019 Apr 15;22(4):e25275. doi: 10.1002/jia2.25275 (PMC6462810; doi:10.1002/jia2.25275)
Supplement: Supplementary file 1 — Table S1. Search strategy: Ovid MEDLINE [file JIA2-22-e25275-s001.docx]

Supplemental Table 1: Search Strategy: Ovid MEDLINE

| OVID Search  (Run on 9/25/2017) |
| --- |
| 1. exp HIV/ (93835) |
| 2. exp HIV Infections/ (267006) |
| 3. exp Anti - HIV Agents/ (63013) |
| 4. Immunodeficiency Virus, Feline/ (1572) |
| 5. exp Feline Acquired Immunodeficiency Syndrome/ (867) |
| 6. exp Simian Immunodeficiency Virus/ (5981) |
| 7. exp Simian Acquired Immunodeficiency Syndrome/ (4202) |
| 8. Maternal-Fetal Exchange/ (29806) |
| 9. Infant Behavior/ (3033) |
| 10. in utero.mp. (23765) |
| 11. Prenatal Exposure Delayed Effects/ (25474) |
| 12. Infectious Disease Transmission, Vertical/ (14429) |
| 13. prenatal.m_titl. (33259) |
| 14. embryo, mammalian/ or embryo, nonmammalian/ (71444) |
| 15. Chimera/ (10023) |
| 16. exp Fetal Diseases/ (66683) |
| 17. exp Pregnancy Complications, Infectious/ (42867) |
| 18. exp fetus/ (155450) |
| 19. Animals, Newborn/ (111448) |
| 20. 8 or 9 or 10 or 11 or 12 or 13 or 14 or 15 or 16 or 17 or 18 or 19 (491432) |
| 21. 1 or 2 or 3 or 4 or 5 or 6 or 7 (318198) |
| 22. 20 and 21 (14394) |
| 23. de.fs. (2831877) |
| 24. to.fs. (397653) |
| 25. tm.fs. (137033) |
| 26. 23 or 24 or 25 (3109980) |
| 27. 22 and 26 (8619) |
| 28. limit 27 to english language (7835) |
| 29. exp Brain Injuries/ (61531) |
| 30. exp Nervous System Diseases/ (2623397) |
| 31. exp Neuropsychological Tests/ (87493) |
| 32. exp v / (224923) |
| 33. 29 or 30 or 31 or 32 (2682568) |
| 34. 28 and 33 (172) |
