# Supplementary material for: In utero exposure to HIV and/or antiretroviral therapy: a systematic review of preclinical and clinical evidence of cognitive outcomes
Source: J Int AIDS Soc. 2019 Apr 15;22(4):e25275. doi: 10.1002/jia2.25275 (PMC6462810; doi:10.1002/jia2.25275)
Supplement: Supplementary file 2 — Table S2. Quality assessment of clinical studies [file JIA2-22-e25275-s002.docx]

Supplemental Table 2: Quality Assessment of Clinical Studies

| Study | Quality Rating |
| --- | --- |
| Jahanshad (2015) | Fair |
| Jankiewicz (2017) | Good |
| Poblano (2004) | Fair-Good |
| Tardieu (2005) | Fair |
| Tran (2016) | Fair-Good |
