# Supplementary material for: In utero exposure to HIV and/or antiretroviral therapy: a systematic review of preclinical and clinical evidence of cognitive outcomes
Source: J Int AIDS Soc. 2019 Apr 15;22(4):e25275. doi: 10.1002/jia2.25275 (PMC6462810; doi:10.1002/jia2.25275)
Supplement: Supplementary file 3 — Table S3. Compliance of preclinical study reporting, by Animal Research: Reporting of In Vivo Experiments (ARRIVE) criteria [file JIA2-22-e25275-s003.docx]

Supplemental Table 3: Compliance of pre-clinical study reporting, by Animal Research: Reporting of *In Vivo* Experiments (ARRIVE) criteria.

| Author | Title/Abstract  reported n / possible n  (% compliant) | Introduction  reported n / possible n  (% compliant) | Methods  reported n / possible n  (% compliant) | Results  reported n / possible n  (% compliant) | Discussion  reported n / possible n  (% compliant) | Total Average  total reported n / total possible n  (% compliant) |
| --- | --- | --- | --- | --- | --- | --- |
| Applewhite- Black (1998) | 2/2  (100) | 3/3  (100) | 7.66/9  (85) | 3/4  (75) | 2.17/3  (72) | 17.83/21  (85) |
| Barks (1993) | 2/2  (100) | 2/3  (67) | 4.75  (53) | 3/4  (75) | 2.17/3  (72) | 13.92/21  (66) |
| Busidan (1999) | 2/2  (100) | 3/3  (100) | 8.1/9  (90) | 3.5/4  (88) | 1.66/3  (56) | 18.26/21  (87) |
| Calamandrei (1999a) | 2/2  (100) | 3/3  (100) | 7/9  (78) | 3.5/4  (87) | 1.66/3  (56) | 17.16/21  (82) |
| Calamandrei (1999b) | 2/2  (100) | 3/3  (100) | 6.66/9  (74) | 2.5/4  63 | 1.33/3  (44) | 15.49/21  (74) |
| Calamandrei (1999c) | 2/2  (100) | 1.5/2  (75) | 6.33/9  (70) | 3/4  (75) | 1.33/3  (44) | 14.16/21  (67) |
| Calamandrei (2000a) | 2/2  (100) | 1.5/2  (75) | 7.66/9  (85) | 2/4  (50) | 1.66/3  (56) | 14.82/21  (71) |
| Calamandrei (2000b) | 2/2  (100) | 1/2  (50) | 6.66/9  (74) | 2/4  (50) | 1.17/3  (39) | 12.83/21  (61) |
| Calamandrei (2002a) | 2/2  (100) | 1/2  (50) | 7.33/9  (82) | 2/4  (50) | 1.66/3  (56) | 13.99/21  (67) |
| Calamandrei (2002b) | 2/2  (100) | 1.5/2  (75) | 3.38/9  (38) | 2/4  (50) | 1.33/3  (44) | 10.21/21  (49) |
| Fitting (2008) | 2/2  (100) | 1.5/2  (75) | 7.33/9  (82) | 2.5/4  (62) | 1/3  (33) | 14.33/21  (68) |
| Levin (2003) | 2/2  (100) | 1/2  (50) | 6.66/9  (74) | 2.5/4  (62) | 1.33/3  (44) | 13.49/21  (64) |
| Melnick (2002) | 2/2  (100) | 1.5/2  (75) | 6.84/9  (76) | 3/4  (75) | 1/3  (33) | 14.34/21  (68) |
| Melnick (2005) | 2/2  (100) | 1.5/2  (75) | 7.15/9  (79) | 3/4  (75) | 1/3  (33) | 14.65/21  (70) |
| Morton (1993) | 1/2  (50) | 1/2  (50) | 2.34/9  (26) | 2.5/4  (62) | 1.33/3  (44) | 8.17/21  (40) |
| Ricceri (2001) | 2/2  (100) | 1.5/2  (75) | 8.17/9  (90) | 2/4  (50) | 1.83/3  (61) | 15.5/21  (74) |
| Rondinini (1999) | 2/2  (100) | 1.5/2  (75) | 7.33/9  (82) | 2/4  (50) | 1.33  (44) | 14.16/21  (67) |
| Venerosi (2001) | 2/2  (100) | 1.5/2  (75) | 6.81/9  (76) | 3/4  (75) | 1.5/3  (50) | 14.81/21  (71) |
| Venerosi (2005) | 2/2  (100) | 1.5/2  (75) | 4.16/9  (46) | 1/4  (25) | 1/3  (33) | 9.66/21  (46) |
| Zuena (2013) | 2/2  (100) | 1.5/2  (75) | 7.33/9  (82) | 2.5/4  (63) | 2.33/3  (78) | 15.66/21  (75) |

Notes: The ARRIVE guidelines were developed in 2010, after 19 of 20 studies included in this review.

Sub-items are given partial credit according to how many other sub-items are scored and how completely they were addressed. For example, if there are 3 sub-items within a question and only one was reported, that item will receive a score of 0.33. If that sub-item was only partially reported, it would only receive 50% of the possible points, so 0.17.
